# Deep convolutional neural networks for mammography: advances, challenges and applications

**DOI:** 10.1186/s12859-019-2823-4

**Published:** 2019-06-06

**Authors:** Dina Abdelhafiz, Clifford Yang, Reda Ammar, Sheida Nabavi

**Affiliations:** 1 Department of Computer Science and Engineering, University of Connecticut, Storrs, 06269 CT USA; 2 The Informatics Research Institute (IRI), City of Scientific Research and Technological Application (SRTA-City), New Borg El-Arab, Egypt; 3 Department of Diagnostic Imaging, University of Connecticut Health Center, Farmington, 06030 CT USA

**Keywords:** Mammograms (MGs), Breast cancer, Deep learning (DL), Convolutional neural networks (CNNs), Machine learning (ML), Transfer learning (TL), Computer-aided detection (CAD), Classification, Feature detection

## Abstract

**Background:**

The limitations of traditional computer-aided detection (CAD) systems for mammography, the extreme importance of early detection of breast cancer and the high impact of the false diagnosis of patients drive researchers to investigate deep learning (DL) methods for mammograms (MGs). Recent breakthroughs in DL, in particular, convolutional neural networks (CNNs) have achieved remarkable advances in the medical fields. Specifically, CNNs are used in mammography for lesion localization and detection, risk assessment, image retrieval, and classification tasks. CNNs also help radiologists providing more accurate diagnosis by delivering precise quantitative analysis of suspicious lesions.

**Results:**

In this survey, we conducted a detailed review of the strengths, limitations, and performance of the most recent CNNs applications in analyzing MG images. It summarizes 83 research studies for applying CNNs on various tasks in mammography. It focuses on finding the best practices used in these research studies to improve the diagnosis accuracy. This survey also provides a deep insight into the architecture of CNNs used for various tasks. Furthermore, it describes the most common publicly available MG repositories and highlights their main features and strengths.

**Conclusions:**

The mammography research community can utilize this survey as a basis for their current and future studies. The given comparison among common publicly available MG repositories guides the community to select the most appropriate database for their application(s). Moreover, this survey lists the best practices that improve the performance of CNNs including the pre-processing of images and the use of multi-view images. In addition, other listed techniques like transfer learning (TL), data augmentation, batch normalization, and dropout are appealing solutions to reduce overfitting and increase the generalization of the CNN models. Finally, this survey identifies the research challenges and directions that require further investigations by the community.

**Electronic supplementary material:**

The online version of this article (10.1186/s12859-019-2823-4) contains supplementary material, which is available to authorized users.

## Background

Breast cancer is the second most common cause of cancer death in women. According to the American cancer society’s latest statistics, it is estimated that 40,610 women in the USA are expected to die in 2017 from breast cancer. As of March 2017, there are more than 3.1 million women with a history of breast cancer in the USA [1]. Mammography is one of the most widely used methods for breast cancer screening and has contributed significantly to the reduction of the mortality rate through early detection of cancer [2]. However, the complexity of mammograms (MGs) and the high volume of exams per radiologist can result in false diagnosis [3, 4].

Computer-aided detection (CAD), which employs image processing techniques and pattern recognition theory, has been introduced to provide an objective view to radiologists [2]. Studies have shown the effectiveness of CAD models; however, accurate detection of breast cancer has remained challenging [2]. Recent studies show that CAD models cannot improve significantly the diagnostic accuracy of mammography [5]. The biggest challenge in using CAD for abnormality detection in MGs is the high false positive rates (FPR). False positives result in patient anxiety, additional radiation exposure, unnecessary biopsies, high callback rates, increased health care costs, and additional assessment [4]. In the USA, millions of women undergo screening mammography each year, as a result, even a small reduction in the FPR result in a widespread benefit [1, 6]. The limitations of current CAD indicate the need for new, more precise detection methods.

Recent advances in computational technologies, significant progress in machine learning and image processing techniques, and prevalence of digital MG images have opened up an opportunity to address the challenging issue of early detection of breast cancer using deep learning (DL) methods [7–10]. Recently, DL methods, specially convolutional neural networks (CNNs, also known as ConvNets) have gained lots of attentions to CAD for MGs as they help overcome CAD systems’ limitations [2, 8, 9, 11]. CNNs achieve higher detection accuracy than CAD models, and help radiologists provide more accurate diagnosis by delivering quantitative analysis of suspicious lesions [10, 12–14]. A recent research study shows that using DL methods drop human error rate for breast cancer diagnoses by 85% [15]. Current CNN models are designed to improve radiologists’ ability to find even the smallest breast cancers at their earliest stages alerting the radiologist to the need for further analysis [12, 15].

Recent studies used CNNs to generate a standard description of lesions, which can help radiologist in making a more accurate decision [12, 14]. Moreover, advances in CNNs can not only aid radiologists, but also eventually make diagnosis systems to read MGs independently in the near future [12]. In the last few years, CNNs have led to breakthroughs in a variety of pattern recognition and classification problems for natural images due to the availability of big data repositories, fast graphical processing units, and the power of parallel and distributed computing [7, 10, 16, 17].

Training a deep CNN model with a limited number of medical data is very challenging, which has been addressed by using transfer learning (TL) and augmentation techniques [7, 16, 18]. Studies show that CNN methods that compare images from left and right breasts [19] and also the craniocaudal (CC) and mediolateral-oblique (MLO) view of each breast can improve the accuracy of detection and reduce the false positives [15, 20–25]. CNNs have also been used in the risk assessment applications to increase the accuracy of early detection breast cancer by radiologist [26–35]. In this work, we summarize almost all contributions, as of November 2017, to the field of DL in MGs, in particular using CNNs.

## Methods

### Criteria for inclusion/exclusion of studies in the survey

**Fig. 1:**
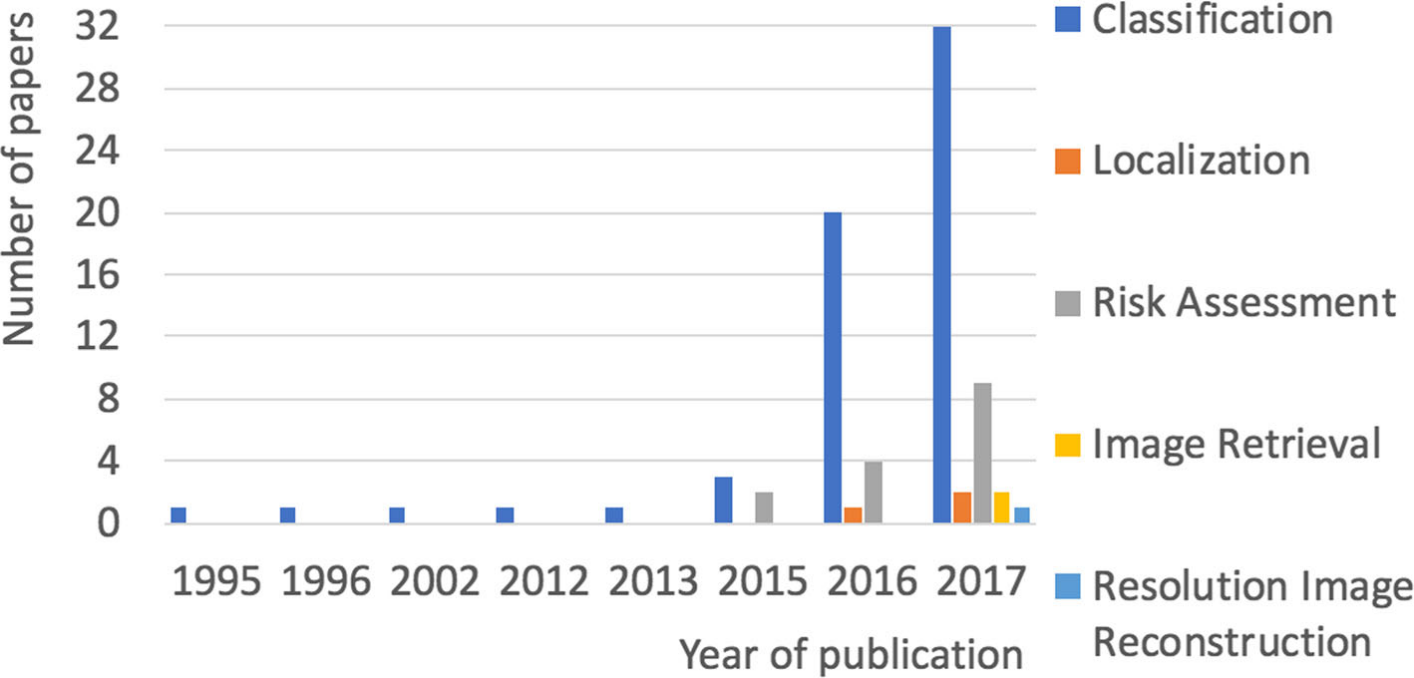
A breakdown of the studies included in this survey in the year of publication grouped by their neural network task. Since 2016 the number of studies on CNN for MGs has increased significantly

We carried out a comprehensive literature research, using the defined keywords given in Table 1, on journals and proceedings of scientific conferences including, but are not limited to the following scientific databases: Scopus, ACM Digital Library, Science Direct, IEEE Explore Digital Library, PubMed, Web of Science. In total, we considered 83 studies from the time period of 1995 to Nov 2017. These studies focus on implementing CNNs for lesion localization and detection, risk assessment, image retrieval, high resolution image reconstruction and classification tasks in MG images. The inclusion/exclusion criteria we used for this review are presented in Table 1. Figure 1, shows a breakdown of the studies included in this survey in the year of publication grouped by their neural network task.

**Table 1:** Inclusion/exclusion for the systematic review

Category	Criteria
Time period	Published from 1995 to the present (Nov 2017).
Databases	Private and public databases.
Publication	English articles in print.
	Excluded articles accepted for publication before appearance in journals or conferences as of Aug 2017.
Research focus	All Implementation of CNNs for breast cancer in Mammography.
Keywords	Deep learning, convolutional neural networks, breast cancer, mammography and transfer learning.
Abnormalities	Mass, calcification, architectural distortion and asymmetries.

In this study, we addressed the following research questions:

- Does this study focus on using a CNN for detecting abnormalities in MGs?
- What is the task of the implemented CNN?
- What are the databases, database size, image resolution, image type, abnormalities involved in the development of the CNN?
- What are the methodologies used for the setup and pre-processing of the data-set?
- Can deep networks perform well on medical images specifically MGs?
- What are the learning methods used for training the CNNs?
- What are the best practices that were applied to increase the accuracy of detection of abnormalities?
- What are the advantages and limitations presented by the methodologies employed in CNNs?
- Is it an end-to-end (E2E) training method?
- Is transfer learning from natural imagery to the medical domain relevant?
- Is combining learned features with hand-crafted features will enhance the accuracy of certain mammographic task?
- What are the common toolkits used in mammography?
- What are the challenges to train deep neural network for mammography data-set?
- How imbalanced data-sets impact the performance of CNNs?
- What is the common cross-validation method used with MGs?
- Which activation functions are commonly used for training MGs?

### Breast cancer digital repositories

Mammographic databases play an important role in training, testing, and evaluation of DL methods. The amount of data needed to train a DL network is massive compared to the data needed to train traditional neural networks. The availability of comprehensive annotated databases is critical for advancing DL development in medical imaging. The most common findings seen on mammography are abnormal areas of mass, calcifications (MCs), architectural distortion (AD), and asymmetries. There are common publicly available databases for MGs: the Mammographic Image Analysis Society (MIAS) database [36], Digital Database for Screening Mammography (DDSM) [19], INbreast database [37], Breast Cancer Digital Repository (BCDR) [38], Image Retrieval in Medical Applications (IRMA) [39].

Table 2 compares the publically available MG databases according to the origin, the number of images, size of images, views (CC, MLO), digital or film database, the format of images, resolution of images, and the distribution of normal, benign and malignant images. Other databases used in literature are private and restricted to individual organizations [21, 26, 27, 31, 34, 40–46]. The public databases present a wide variability of patients’ cases and a mixture of normal, benign, and malignant cases. Annotations include the location and boundaries of the lesions performed by imaging specialists. The public repositories have collected film screen MGs (FSMs) [36, 38, 39], and/or digital mammography (FFDM) [37–39, 47] with different resolutions. Digital MG images are usually saved in the DICOM format that gathers not only the image but also some related meta-data as in [37, 38, 47]; however, some databases use different formats [36, 38, 39, 48].

**Table 2:** Comparison between widely used databases in literature respect to size of images, views (CC, MLO), digital or film databases, the format of images, bits/pixel (bpp) and the distribution of normal, benign and malignant images

Database	Image-size	Views	Type	Format	bpp	#Normal	#Benign	#Malignant
DDSM	3118 ×5001	Both	FSM	LJPEG	12	914	870	695
IRMA	Several	Both	Both	PNG	12	1108	1284	1284
INbreast	Several	Both	FFDM	DICOM	16	67	220	49
MIAS	1024 ×1024	MLO	FSM	PGM	8	207	69	56
BCDR-F01	720 ×1168	Both	FSM	TIF	8	0	187	175
BCDR-F02	720 ×1168	Both	FSM	TIF	8	0	426	90
BCDR-F03	720 ×1168	Both	FSM	TIF	8	0	426	310
BCDR-D01	Several	Both	FFDM	DICOM	14	0	85	58
BCDR-D02	Several	Both	FFDM	DICOM	14	0	405	51
BCDR-DN01	Several	Both	FFDM	DICOM	14	200	0	0

The images of the MIAS database are of low resolution and have strong noise. The MIAS database is an old database that contains a limited number of images. Despite all these drawbacks, it has been widely used in literature until now [49–51]. DDSM is a huge repository used in many studies [23, 24, 32, 49, 52–65]. DDSM images are saved in non-standard compression files that require use of decompression codes. Moreover, the Region of Interest (ROI) annotations for the abnormalities in the DDSM images indicate general position of lesions, without precise segmentation of them. The IRMA project is a combination of a number of databases of different resolution and sizes. The ROI annotations for these databases are more precise making them more accurate for supervised DL methods. The INbreast database is gaining more attention nowadays and used in [25, 32, 57, 66–70]. Its advantages are high resolution and accurate segmentation of lesions; however, its small size and the limited shape variations of the mass are its drawbacks. BCDR is a promising database but still is in its development phase. BCDR has been used in few studies [71–74]. The strengths and limitations of these databases are summarized in Table 3.

**Table 3:** A summary for the strengths and limitations of the DDSM, IRMA, INbreast, MIAS and BCDR databases

Database	Strength	Limitation
DDSM	Big widely used database.	Non-standard format.
	Shape variations of different lesions.	Not precise position of lesions.
IRMA	Accurate position of lesions.	Non-standard format.
	High resolution.	
INbreast	Accurate position of lesions.	Limited size.
		Limited mass shape variations.
	Standard file format.	
		Old database.
		No more supported.
MIAS	Still widely used.	Limited size.
		Images are of low resolution.
		Has MLO view only.
	Different resolutions.	
BCDR	Accurate position of lesions.	Limited size.
	Standard file format.	
	Still in their development phase.	

### Convolutional neural networks

In fact, DL is not a new idea, which even dates back to 1940s [7, 75] for medical images. Shallow layer CNNs were used to investigate breast cancer in 1995 [40, 76]. Famous CNNs such as Alex-Net [16], ZF-Net [77], GoogLeNet [78], VGG-Net [79] and ResNet [80] have brought about breakthroughs in processing images. Alex-Net architecture is extensively used in medical imaging for breast cancer detection. DL is a subset of machine learning that requires a huge number of labeled data to train the models. The term “deep” usually indicates the number of hidden layers in neural networks, e.g. ResNet has a depth of 152 layer which is 8 × deeper than VGG-Net. Since 2012, CNNs have become more popular and have attracted more attention because of the increasing computing power, availability of lower cost hardware, open source algorithms, and the rise of big data [16].

**Fig. 2:**
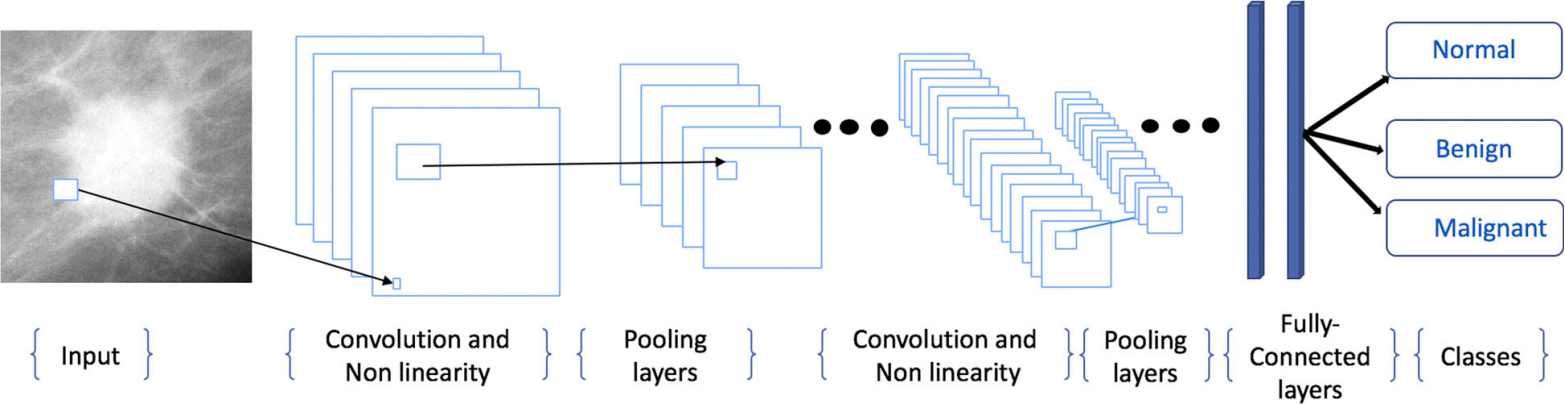
The CNN architecture is a stack of Convolutional layer (Conv), Nonlinear layer (e.g. ReLU), Pooling layer (Pool), and a Loss function (e.g. SVM/Softmax) on the last (Fully connected) layer. The output can be a single class (e.g. Normal, Benign, Malignant)

The structure of CNNs is very similar to that of ordinary neural networks. The basic CNN architecture is a stack of convolutional layer (Conv), nonlinear layer (e.g. ReLU), pooling layer (e.g. Max-pooling), and a loss function (e.g. SVM/Softmax) on the last fully connected (FC) (Fig. 2). The output can be a single class (e.g. normal, benign, malignant) or a probability of classes that best describes the image. The input to a convolutional layer is a W1 ×H1 ×D1 image where W1 is the width and H1 is the height of the image and D1 is the number of channels, e.g. an RGB image has D1=3. The convolutional layer will have F filters (e.g. 12 filters) of size N ×*N*×D1 where N is smaller than the dimension of the image and D1 is the same as the number of channels (e.g. 5 ×5×3 (i.e. 5 pixels width and height, and 3 because images have depth 3, the color channels).

During the convolution operation, each of the F filters convolves with the image to produce K feature maps of volume size W2 ×H2 ×D2 where: W2=H2=(W1-F+2P) /S+1, S is the number of strides, D2=F, and P is the amount of zero padding. For each feature map, a non-linear activation function is applied (e.g. ReLU). A non-linear activation function leaves the size of the volume unchanged (W2 ×H2 ×D2). After applying ReLU, a down-sampling operation called Pool is applied along the spatial dimensions (width, height) of the result feature map. After pooling, there may be any number of fully connected layers that compute the class scores (Fig. 2). More details about the architecture of CNNs can be found in [16, 81].

### Popular CNNs

Alex-Net [16], ZF-Net [77], GoogLeNet [78], VGG-Net [79] and ResNet [80] have been extensively used as a pre-trained networks to classify images for medical domains instead of training a network from scratch. Table 4 shows the configurations of the most popular CNNs. Generally, training a deep CNN requires extensive computational and memory resources. Training these networks from scratch typically takes days or weeks on modern GPUs (Table 4). All these networks were trained on the 1000 object category classification on the ImageNet data-set [82]. The ImageNet data-set consists of a 1.2M image training set, a 50K image validation set, and a 100K image test set. Two error rates are reported for these networks: top-1 and top-5, where the top-5 error rate is the fraction of test images for which the correct label is not among the five labels considered most probable by the model. All these network architectures use the data augmentation technique to prevent overfitting with dropout initially set to 0.5.

**Table 4:** The configurations of AlexNET, ZF-NET, GoogLeNET, VGG-NET and ResNET models

	AlexNet [16]	ZF-Net [77]	GoogLeNet [78]	VGG-Net [79]	ResNet [80]
Year	2012	2013	2014	2014	2015
Image Resolution	227 ×227	227 ×227	224 ×224	224 ×224	2244 ×224
Number of layers	8	8	22	19	152
Number of Conv-Pool layers	5	5	21	16	151
Number of FC layers	3	3	1	3	1
Full connected layer size	4096,4096,1000	4096,4096,1000	1000	4096,4096,1000	1000
Filter Sizes	3, 5, 11	3, 5, 11	1,3,5,7	3	1,3,7
Number of Filters	96 - 384	96 - 384	64 - 384	64 - 512	64 - 2048
Strides	1, 4	1, 4	1, 2	1	1, 2
Data Augmentation	+	+	+	+	+
Dropout	+	+	+	+	+
Batch Normalization	-	-	-	-	+
Number of GPU	2 GTX	1 GTX	A few high-end	4 Nvidia	
580 GPUs	580 GPUs	GPUs	Titan Black GPUs	Titan Black GPUs	8 GPUs
Training Time	5:6 days	12 days	1 week	2:3 weeks	2:3 weeks
Top-5 error	16.40%	11.2%	6.70%	7.30%	3.57%

Alex-Net [16] was the first CNN to win the ImageNet Challenge in 2012. AlexNet’s CNN consists of five Conv layers and three fully connected (FC) layers. Within each Conv layer, there are 96 to 384 filters and the filter size 3 ×3, 5 ×5, 11 ×11, with 3 to 256 channels each. A ReLU non-linearity is used in each layer. Max-pooling of 3 ×3 is applied to the outputs of layers 1, 2 and 5. Alex-Net used a stride of 4 at the first layer of the network. AlexNet’s model requires 61M weights to process one 227 ×227 input image (top-5 error of 16.40%). ZF-Net [77] is a slightly modified version of Alex-Net model and uses an interesting way of visualizing their feature maps. In ZF-Net, the used visualization technique give insight into the function of intermediate feature layers and the operation of the used classifier. The VGG-Net [79] model reinforces that the CNNs have to have a deep network of layers. GoogLeNet [78] has 22 layers. It introduced an inception module to the CNN model. It has pieces of the network that are working in parallel in contrast to previous CNN models, which have only a single serial connection. ResNet [80], also known as Residual Net, uses residual connections to go even deeper. ResNet determines an object’s exact location, which is a huge jump in CNNs. ResNet is 8 × deeper than VGG-Net with lower complexity. The ResNet with 152 layers was the winner of the ImageNet challenge 2015 [82] (top-5 error of 3.57%). it has 60M weights. YOLO is another famous CNN named that is recently used for object classification and localization while processing the image only once, as is implied by it’s name, You Only Look Once [83, 84]. Table 4 shows that the number of layers are going deeper and deeper within the newer implementations as in ResNet.

## Results

### CNNs best practices

In this section, we explain the practices that contribute to improve the performance of CNNs for MGs. It goes beyond the scope of this paper to discuss all the best practices done in CNNs in general, but we are going to highlight and focus on some of them that show significant changes in the classification accuracy when applied to MG images. Recent survey papers [7, 8, 85] discuss more trends for natural images.

#### Data preparation

**Pre-processing of MG images**

Pre-processing of MG images is an essential task before training CNNs [63, 66, 71, 72, 86]. The pre-processing consists of contrast enhancement, noise removal, and breast segmentation. Breast segmentation includes the remove of the background area, labels, artifacts, and pectoral muscle which disturb the detection of Mass/MCs [45, 50]. It is important to have good separation between foreground and background pixels and do not remove the important information in images [59, 87, 88]. The commonly used filters for image enhancement and noise reduction are the adaptive mean filter, median filter, and contrast limited adaptive histogram equalization (CLAHE) [62, 89–92].

**Image size, cropping, and down-sampling**

Most studies have used segmented ROIs in order to reduce the computation of the CNNs and to avoid the issue of small training data. These ROIs can be obtained by a manual segmentation of the images using the available ground truth data, or an automatic detection system. The ROIs are cropped and re-scaled to r ×r pixels with the lesion centered within the image. However, using very small subsampled (e.g. 32 ×32) patches may not contain enough detail to improve the classification results as in [40, 41, 44, 63, 66, 67, 70, 74, 93].

Two strategies have been utilized to use full image size for training CNNs on MGs instead of ROIs. The first strategy, down-sample high resolution images to ≈250×250. However, the requirement to find small mass regions or MCs clusters in down-sampled high resolution images is unlikely to be successful for MGs [65]. The second strategy, train a patch-level CNN classifier, which is then used as feature extractor to an image-level model. In the image-level model, each image is partitioned into a set of patches with a minimal overlap such that each patch is contained entirely within the image. Final classification involves aggregation across patches and the CC & MLO views [65].

**Mixing databases**

In literature, researchers mix several databases to analysis their CNNs. The fusion from different image type (FSM and FFDM) assists CNNs in term of detection rate. Researchers in [32, 49, 51, 52, 57, 94, 95] compared both image quality and detection on FFDM and FSM databases. They have shown that a CNN using FFDM images gives better detection rate than using FSM images. Moreover, these studies show that DL training using the fusion of both FFDM and FSM lower the number of false detections [93, 94].

**Learned and hand-crafted features**

The hand-crafted features (i.e. Haar-like features, histogram of oriented gradients (HOG), and histogram of the gradient divergence (HGD)) are commonly used with traditional machine learning approaches for object recognition like support vector machines. CNNs are able to extract features from the input image data-sets. Thus, CNNs remove the necessity of the time-consuming hand-crafted features.

However, the authors in [21, 23, 54, 71, 87, 96–100] have demonstrated the importance of combining the extracted features using deep CNNs with hand-crafted features like texture, and shape. Interestingly, the combination of both representations (learned and hand-crafted features) resulted in a better descriptor for Mass/MCs lesion classification [71, 100]. The reason behind using hand-crafted features is that the learning process should be guided by a training data-set that has a wide variability of texture and shape features. For example, Dhungel et al. [97] proposed two-step training process involving pre-training based on a large set of hand-crafted features. The second stage fine-tunes the features learned in the first stage to become more specialized for the classification problem.

Using hand-crafted features depends on the size of the data-set. With small training data-set, generating hand-crafted images could result in a better model for Mass/MCs lesion classification. Also, employing some hand-crafted features that specifically target small and missed lesions is a more effective strategy than adding extra cases to the train a data-set. Thus, the performance of CNNs trained with small data-set can be improved by incorporating hand-specified features to deal with cases that cause false positives or false negatives [23].

#### Hyper-parameters

Hyper-parameters are variables which determine the network structure (e.g. number of hidden layers), and the variables which determine how the network is trained (e.g. learning rate). Hyper-parameters are manually chosen before training the CNNs.

**Data augmentation**

Data augmentation is an appealing solution to reduce overfitting and increase the generalization of the model and boost the performance. Overfitting happens in CNNs when the models learn too well the details from training data, but they do not generalize well from the training data, in order to make good predictions about the future unseen data. As a result, the performance of the trained model is poor for testing data. That usually happens when the size of training data-set is too small compared with the number of model parameters that need to be learned.

Data augmentation artificially creates new sample images by applying transformations like flipping, and rotation to the actual data. Common data augmentation techniques for mammography images are horizontally flipping, rotations (90, 180, and 270 degrees), jittering, and random scaling. Such data augmentation generates relevant training samples because tumors may present in various orientations and sizes. Thus, augmentation techniques do not change the underlying pathology of the masses. Data augmentation has been employed by many studies [12, 22, 23, 30, 33, 34, 40–45, 50, 53–55, 57, 58, 63, 66, 70–73, 96, 97, 101–110].

**Going deeper**

In CNN, the design of the network architecture completely depends on the model requirements and the size of the data-set. The CNNs in [53, 66, 96] have a fewer number of layers but show good accuracy. However, the work done in [63, 72, 73, 78] shows that we can get better performance in term of higher area under the ROC curve (AUC) as the architecture goes deeper and trained on more data. Deep architectures can lead to abstract representations because more abstract shapes can often be constructed in terms of less abstract ones captured in earlier layers. Adding more layers will help the model to extract more features. But adding more layers can be done to a certain extent and there is a limit. After that, instead of extracting features, it results in overfitting the network that can lead to false positives. Adding more hidden layers will promote the accuracy for large data-sets. Adding layers unnecessarily to a CNN will increase the number of parameters, and for a smaller data-set, it will reduce accuracy of the test data. Deep architectures are often challenging to train effectively, and this has been the subject of more recent research. Choosing a smaller network or a larger one cannot be estimated theoretically. A trade-off between accuracy and deep networks need to be done with trial and error method and some experience and practice on the basis of the data-set.

**Learning rate**

Learning rate (LR) is one of the most important hyper-parameters, which influences the CNNs’ performance. Deep learning models are typically trained by a stochastic gradient descent optimizer. There are many variations of stochastic gradient descent as Adam, RMS Prop, Adagrad, etc. All these optimizers let users set the learning rate. Learning rate controls how much the network parameters are adjusted in order to minimize the network’s loss function. If the LR is too small, the CNN will converge after many iterations to the best values. However, if LR is too high, it can cause undesirable divergent behavior in the loss function. Famous learning rate policies are step decay, quadratic decay, square root decay and linear decay [85]. A common practice when dealing with MG images, is to use a step decay rate where the LR is reduced by some percentage after a set number of training epochs. For example, Yi et al. [23] used a learning rate of 0.001 with decay rate of 0.99 per epoch, and a regularization coefficient of 10^−5^ for training their CNN. Another common practice is to use a small learning rate (e.g. 0.001) to train a pre-trained network, since we expect well-adjusted pre-trained weights compared to randomly initialized weights.

**Activation functions**

Recently, many variations of rectified linear unit (ReLU) function have been proposed for activation function such as leaky ReLU, parametric ReLU, and randomized ReLU [111]. There are other popular activation functions such as sigmoid, and tanh. The activation functions bring non-linearity into CNNs. Sigmoid presents a serious disadvantage called the vanishing gradient problem. In the vanishing gradient problem, the gradient of small input values to sigmoid functions tends to get smaller (close to zero) as gradients are computed backward through the hidden layers, resulting in slow learning in the earlier layers of the model. Slow learning is highly avoided in DL since it results in expensive and tedious computations [112].

ReLU became a popular choice in DL and even nowadays provides outstanding results as it solves the vanishing gradient problem [111]. ReLU has gradient one for positive inputs and zero for negative inputs. As long as values are above zero, the gradient of the activation function will be one, meaning that it can learn anyways. This solves the vanishing gradient problem present in the sigmoid activation function. On the downside, once the gradient is zero the corresponding nodes do not have any influence on the network anymore, which is known as “dying ReLU” problem. Leaky ReLU is one attempt to overcome the dying ReLU problem [113]. Instead of the output of ReLU being zero when input is less than zero, a leaky ReLU will provide a small negative slope (*α* of 0.01, or so). This small slop reduces the sparsity but, on the other hand, makes the gradient more robust for optimization, since in this case, the weight will be adjusted for those nodes that were not active with ReLU. When the slop is not constant (e.g. 0.01) then it is called randomized ReLU.

A detailed explanation of the advantages and disadvantages of different activation functions are discussed in [16, 111, 112]. Theoretically, leaky ReLU is in general better than ReLU. However, ReLU has been chosen as an activation function in most of the CNNs for MGs as it allows faster learning [58, 64, 65, 70, 114, 115].

#### Techniques for improving the CNNs performance

**Dropout**

Dropout is a regularization technique proposed in [116] that superior the other regularization methods (L1, L2, Max norm). Dropout prevents a CNN model from overfitting. This technique randomly selects neurons and ignore them during training. They are “dropped-out” randomly. This means that their contribution to the activation of downstream neurons is temporally removed on the forward pass and any weight updates are not applied to these neurons on the backward pass [16]. Smirnov [117] has shown a comparison of regularization methods with deep CNNs and showed that the dropout technique is in general better than other regularization techniques. The authors in [12, 22, 25, 44, 58, 70, 73, 94, 106, 106, 108] have used dropout in their work with MGs. The dropout of 0.5 is a common value for mammography images.

**Batch normalization**

In a CNN model, a batch normalization (BN) layer normalizes input variables across a mini-batch (a subset of the training data-set). First, the BN layer normalizes the activations of each channel by subtracting the mini-batch mean and dividing by the mini-batch standard deviation. Then, the BN layer shifts the input by a learnable offset *β* and scales it by a learnable scale factor *γ*, thus reduces the networks’ internal covariant shift. BN speeds up training of CNNs and reduce the sensitivity to network initialization. According to [118], BN allows the use of much higher learning rates and less care about initialization as it acts a regularize. BN results in faster convergence and as a consequence overall faster training for a CNN. Besides that, BN regulates the values going into each activation function. With BN, saturating nonlinear activation functions (e.g. sigmoid) that do not work well in deep networks tend to become viable again. Similar to dropout, BN adds some noise to each hidden layer’s activations. Therefore, using BN causes less dropout value. BN has been used in CNNs for MG images [65, 73, 101]. For mammography, it is recommended to not depend only on BN for regularization; and to use it together with dropout.

**Transfer learning**

Training a deep CNN requires large amounts of labeled training data [11]. Only few studies train an entire CNNs from scratch with random initialization; and the rest use TL approaches either fine-tune a pre-trained network [46, 52, 53, 58, 60, 63, 72, 73, 94, 110, 119, 120] or use a pre-trained network as feature extractor [15, 32, 46, 70]. Recent overviews of TL in deep network models are given in [37, 45, 46, 65]. The need for TL in medical domain occurs because data are scarce and expensive, they are not publicly available, and it is time-consuming to collect and label them by professional radiologists [17, 46, 55, 121–124]. Moreover, training a deep CNN requires extensive computational and memory resources [16, 17, 78].

References [60, 77, 125] show that the main power of a CNN lies in its deep architecture. Extracted features of earlier layers of a pre-trained CNN (i.e. on natural images) contain more generic features (e.g. edge detectors or blob detectors) that are useful for many tasks; but in later layers, generic features are combined and become more specific to the details of the classes contained in the training data-set. Thus, a deep CNN allows extracting a set of discriminating features at multiple levels of abstraction which can be transferable from one domain to another. However, the required level of fine-tuning differs from one application to another. Tajbakhsh et al. [125] show that neither shallow tuning nor deep tuning may be the optimal choice for a particular application. Moreover, layer-wise fine-tuning may offer a practical way to reach the best performance for a certain application and should be chosen experimentally. In addition, the work in [21, 106, 109] has achieved a good performance on a small data-set by pre-training the network on a large data-set of general medical images.

Most of the studies employed TL have used ImageNet’ data-set [82] for pre-training their network [46, 58, 60, 72, 94, 95, 110, 126–128]. The commonly used pre-trained CNNs architectures for mammography are Alex-Net [46, 50, 58, 60, 72, 94, 95, 110, 127, 128], VGG16 [50, 127, 129], ResNet50 [127, 129] and GoogLeNet [58, 72, 127]. All the deep CNN architectures that are pre-trained using ImageNet are designed for a 1000-class classification task. To adapt them to the task at hand, the last three layers are removed from each network and a three new layers (FC layer, soft-max layer, and classification layer) are appended to the remaining structure of each network.

Until large-scale medical image data-sets for mammography became available, the combination of TL and data augmentation is a very promising approach for training deep CNNs. By visualizing the features learned at different layers during the training process, a model can be monitored to closely observe and track its performance [23]. Learned features can indicate whether a model is successfully learning or not, allowing a user to stop the training process early [130].

**Cross-validation**

Cross-validation is a statistical technique to evaluate predictive models by partitioning the original samples into a training set to train the model, and a test set to evaluate it. There are three common types used in literature for validation, the hold-out splits [76, 131], three-way data splits [8, 22, 58, 65, 96], the K-fold cross-validation [20, 23, 25, 26, 49, 94, 110, 115, 132, 133]. In the hold-out data splits, data is split into training set and test set (e.g. 80%, and 20%, respectively). The training set is used to train the model and the test set is used to estimate the error rate of the trained model. In the three-way data splits, data are randomly split into training, validation and testing sets. The CNN model is trained on the training set and is evaluated on the validation set. Training and validation may be iterated a few times till the best model is found. The final model is assessed using the test set.

In the K-folds cross validation, data are split into k different subsets (or folds). The cross-validation process is repeated K times (the folds), with each of the K sets used exactly once as the test set. The K error estimates from the folds can then be averaged to produce a single estimation. Cross-validation avoids overfitting and gives a less biased estimate of the performance of the model [67, 134]. In practice, the choice of the number of folds depends on the size of the data-set. In literature, the common strategy is to use K-fold cross validation for mammography. For large data-sets, it is a common choice to use 3 to 5-fold cross-validation. For small mammography data-set, it is a common choice to use 10-fold cross-validation.

**Context and patient information**

Integrating some information such as patient age, breast density and other context like the view type (CC or MLO) into a CNN method can improve the detection rate of CNNs [96]. Multi-modal machine learning aims to build models that can process and relate information from multiple modalities (e.g. images and text) with a score level fusion at the final prediction results.

**Multi-view and single-view images**

It is a good practice to use both CC & MLO views to detect abnormalities. A true abnormality can usually be detected on two different views of a MG. Recent studies in [15, 20–25, 95, 107] lead to significant improvements of multi-view (MV) approaches compared to single-view (SV) ones, demonstrating that the high-level features of the individual CNN models provide a robust representation of the input images. Comparing two views can aid in the reduction of false positives and false negatives.

**Balanced and imbalance distribution**

A couple of publicly available databases (e.g. INbreast, DDSM) are constructed to include approximately the same proportions of normal and abnormal cases, which is a balanced distribution of classes. Other databases called imbalance distribution (natural distribution) databases, which include unequal proportions of normal and abnormal cases. Training CNN models directly on imbalanced data-sets may bias the prediction towards the more common classes like normal, resulting in false negatives. Whereas the minority ones are misclassified frequently [135]. The authors in [20, 21, 32, 45, 46, 53, 56, 58, 73, 74] have pointed out that the balance of the number of samples per class has a great impact on the performance of the system. However, the authors in [22, 44, 96] used a natural distribution databases. According to [136] choosing a wrong distribution or objective function while developing a classification model can introduce bias towards potentially uninteresting class (non-cancerous). For MG images, it is preferable to use a balanced data-set. Different approaches to handle imbalanced data-sets include random under-sampling and random over-sampling techniques [135]. Random under-sampling aims to balance class distribution by randomly eliminating majority class samples (normal cases). This is done until the majority and minority class instances are balanced out as done in [74]. In the other side, over-sampling increases the number of instances in the minority class (abnormal cases) by randomly replicating them in order to present a higher representation of the minority class. Unlike under-sampling, over-sampling leads to no information loss.

The appropriate approach (random under-sampling or random over-sampling) depends on the amount of available data-set and the specific problem at hand. Researchers empirically test each approach and select the one that gives them the best results. In the case of using an imbalance data-set, accuracy is not a right metric to evaluate the performance of the model. There are more appropriate scores when using imbalanced data-sets such as F1-score [136] that combines the trade-offs of precision and recall, and outputs a single number reflecting the goodness of a classifier.

**Multi-stage and end-to-end (E2E) methods**

A multi-stage pipeline used for detection and classification of a lesion consists of multiple stages such as pre-processing, image segmentation, feature detection, feature selection and classification stages [137, 138]. End-to-end (E2E) deep learning methods take all these multiple stages and replace it with just a single neural network. Researchers in [12, 15, 24, 40–42, 60, 67, 102, 105] have used one or more stages of this multi-stage pipeline in their CNN systems. In their multi-stage method, a CNN is trained to determine whether a small patch has Mass and/or MCs. Other researchers focused on training a deep CNN for classifying a small ROI or full image into benign or malignant, assuming an existing Mass/MCs detection system as in [23, 25, 43, 50, 55, 56, 62, 66, 71, 72, 87, 104]. In multi-stage methods for CNNs, several cascaded classifiers are trained independently, each classifier makes a prediction, and all predictions are combined into one using different strategies. Dhungel et al. has found that the multi-stage methods are effective in the reduction of false positive detection [97]. Moreover, researchers in [22, 25, 30, 45, 57, 96, 98, 107] used the E2E methods.

E2E methods for MGs are better than multi-stage method when training a CNN with a large data-set. But if the data-set is small in size, then the learning algorithm cannot capture much insight from data. Excluding potentially useful hand-crafted features that are very helpful if well designed is the downside of the E2E approaches. Therefore, the key parameter to choose using E2E deep learning approach is having sufficient data to learn the model.

### Toolkits and libraries for deep learning

Implementing a DL network from scratch is an exhausting process and probably beyond the skills of most medical imaging researchers. It is much more efficient to utilize the publicity available resources. Some criteria should be considered while choosing a library and toolkit including its programming language for interface, the quality of documentation of the toolkit, the ease of programming, the runtime to do thousands of calculations per pixel, the training speed, GPU support for faster performance [17], and lastly its popularity among experts. Recent surveys done in [139, 140] discus the most famous and recent toolkits and libraries used generally for DL. The common toolkits used in training CNNs for mammography are Tensorflow [141], Keras, Caffe [142, 143], PyTorch [144] and MatConvNet [145]. Table 5 gives a comparison between these libraries and their ranking based on the forks received by the community on GitHub.

**Table 5:** A comparison between most famous toolkits and libraries for training mammography

	Interface	Languages	Open source	CUDA support	Pre-trained models	Forks (Github)	Contributions (Github)
TensorFlow	Python	C++, Python	Yes	Yes	Yes	63,603	1,481
Keras	Python, R	Python	Yes	Yes	Yes	11,203	681
Cafee	Python, Matlab, C++	C++, Python	Yes	Yes	Yes	14,868	267
PyTorch	Python	C, Python, CUDA	Yes	Yes	Yes	3,592	644
MatConvNet	Matlab	CUDA	Yes	Yes	Yes	651	24

**Tensorflow** is one of the most popular DL libraries, it was developed by the Google Brain team and open-sourced in 2015 [141]. Tensorflow is a Python-based library capable of running on multiple CPUs and GPUs. It can be used directly to create deep learning models, or by using wrapper libraries (e.g. Keras) on top of it. Tensorflow does not contain many pre-trained models and there’s no support for external data-sets, like Caffe. The framework is written in C++ and Python and has large amount of available documentation. As of today it is the most commonly used deep learning framework.

**Keras** is a very lightweight open source library, easy to use, and pretty straightforward to learn. It was built as a simplified interface for building efficient deep neural networks in just a few lines of code and use Tensorflow as back-end.

**Caffe** is one of the first deep learning libraries developed mainly by Berkeley vision and learning center (BVLC) [142, 143]. It is a C++ library which also has a Python interface and finds its primary application in modeling CNNs. Caffe provides a number of pre-trained networks directly from the Caffe Model Zoo, available for immediate use.

**PyTorch** is a Python library enabling GPU accelerated tensor computation, similar to NumPy. A few advantages of using PyTorch are it’s multi-GPU support, dynamic computational graphs, custom data loaders, optimization of tasks, and memory managements. PyTorch provides a rich API for neural network applications [144]. PyTorch is used by many companies such as Twitter, Facebook and Nvidia to train DL models.

**MATLAB** has a neural network toolbox that provides algorithms to create, train, visualize deep neural networks. TL can be done with pre-trained deep CNNs models (including Inception-v3, ResNet-50, ResNet-101, GoogLeNet, Alex-Net, VGG-16, and VGG-19) and models imported from Keras or Caffe. MATLAB allows computations and data distribution a across multi-core processors and GPUs with the parallel computing toolbox. MatConvNet [145] is an open source implementation of CNNs with a deep integration in the MATLAB environment.

### Applications of deep CNNs for mammography

After describing deep CNNs in the previous section, and different practices that are famous for mammography, we will now turn our focus to how these are used for recognition purposes for mammography. More specifically, we review recent deep CNNs’ applications in mammography such as classification, localization, image retrieval, high resolution image reconstruction and risk analysis. We summarized these recent works in Additional file 1: Table S1.

#### Lesion classifications and detection

The detection of lesions in mammography is a common task for CNNs. In contrast to lesion detection, classification of MGs into benign and malignant is a challenging task that many studies try to address it. The authors in [12, 15, 20, 21, 24, 40, 41, 44, 52, 60, 67, 70, 90, 93, 102, 105, 120] are interested in lesion classification into two classes. They developed a CNN to predict a probability of being normal (NL), contain mass and/or MCs. The studies in [23, 43, 46, 49, 50, 55, 56, 58, 59, 62, 63, 66, 69, 71–73, 94, 99, 100, 104, 132, 146–150] present deep CNN methods to classify the MG images into 2 classes (benign or malign), or three classes (benign, malign or without tumor). The authors in [32, 95] studied the development of malignancy of mass(es). The authors in [15, 40, 42, 44, 151] are interested in the classification and detection of MCs in mammography. Chan et al. [40] introduced one of the earliest application of CNNs to detect clustered MCs. The authors applied enhancement filters for noise reduction on fifty-two FSM images. They observed that the shape of MCs in the breast is randomly oriented, thus they introduced an augmentation technique. Sahiner et al. [41] demonstrated the great effect of mixing CNN representation features and textural features (AUC of 0.873). Lo et al. [102] introduced a multiple circular path CNN coupled with morphological features of ROIs (AUC of 0.89). Sharma et al. [59] extracted geometrical features from MG images and used it with the representation features of their CNN. Their work demonstrates that DL methods are superior to traditional classifiers. Domingues et al. [67] used a shallow CNN that did not outperform traditional CAD methods, as they used a very small data-set to train their network and the selected normal ROIs did not represent every possible aspect of healthy breast tissue. Antropova et al. [100] developed a system incorporating both deep CNN and conventional CAD methods that performed statistically better than either one separately.

Sert et al. [63] stated that human level recall performance in detecting breast cancer considering MCs from MGs has a recall value between 74.5% and 92.3%. In [63], the authors reached a recall value of 94.0% above human level performance. Wang et al. [15] showed that breast arterial calcifications (BACs), detected in MGs, can be useful for identifying risk markers for having cancer. The authors in [15] showed that their CNN method achieves a level of detection similar to the human experts. Kooi et al. [12] employed a deep CNN with a large augmented data-set. Similar to the work of [15], the network in [12] performs similar to experienced radiologists, achieving AUC of 0.87 while the mean AUC of the experienced radiologists is 0.84. In [96], Kooi et al. proposed to use a random forest classifier for mass detection followed by a deep CNN that classifies each detected mass. Their method relies on a manually extracted features and features extracted from CNN layers. In [96], Kooi et al. trained their model on a large data-set and integrated additional information such as lesion location and patient information. Kooi et al. [149] following their work in [12, 96] employed a conditional random field (CRF) that is trained on top of CNN to model contextual interactions such as the presence of other suspicious regions. In [21], Kooi et al. employed a deep MV CNN using a pre-trained network on medical domain. They combined the extracted features using the deep CNN with hand-crafted features.

The studies in [46, 50, 58, 64, 72, 94, 106, 109, 120] demonstrated the use of TL in their work. The authors in [46, 50, 72] showed that CNNs in addition to TL can superior current CAD methods for tumor detection and classification based on small data-sets. Samala et al. [106] demonstrated that MGs can be useful for pre-training a deep CNN for mass detection in digital breast tomosynthesis (DBT). The similarity between masses in mammography and DBT can be observed from the ability of the DCNN in recognizing masses in DBT. In [94], Samala et al. demonstrated that CNNs with TL achieve better generalization to unknown cases than networks without TL. Similar to [94, 106], Hadad et al. [109] described a TL approach for using a pre-trained deep CNN on MGs to improve the detection accuracy of fine-tuned CNN on breast MRI lesions. Suzuki et al. [120] developed a deep CNN pre-trained on natural images, then the authors modified the last fully connected layer and subsequently train the modified CNN using 1,656 ROIs. Similar to [120], Jiao et al. [55] achieved an accuracy of 96.7% by applying fine-tuning on a pre-trained CNN on natural images to extract features for the next procedures. Jiao et al. [64] following his work in [55] proposed metric learning layers to further improve performance of the deep structure and distinguish malignant instances from benign ones. Levy and Jain [58] demonstrated that a fine-tuned pre-trained network significantly outperforms shallow CNNs.

Abbas [49] used speed-up robust features and local binary pattern variance descriptors that are extracted from ROIs. After that, they constructed deep invariant features in supervised and unsupervised fashions through a multilayer CNN architecture. Valvano et al. [151] achieved accuracy of 83.7% for MCs detection using a deep CNN. Jamieson et al. [43] introduced a four-layer unsupervised adaptive deconvolution network to learn the image representation using 739 FFDM images. Sun et al. [105] developed a graph-based semi-supervised learning (SSL) method using a deep CNN, their method allows the users to include the unlabeled data into the DL training data-set. In contrast, Arevalo et al. [69] used supervised training in their method using ROIs annotated manually made by expert radiologists, achieving AUC of 0.86. Arevalo et al. [71] following their work in [69], used a hybrid supervised CNN classifier along with an extensive enhancement pre-processing process. Dubrovina et al. [104] presented a supervised CNN for region classification into semantically coherent tissues. The authors overcame the difficulty involved in a medium-size database by training the CNN in an overlapping patch-wise manner. Teare et al. [62] proposed dual supervised CNNs for classifying full MG images to normal, benign and malignant classes. In their work a random forest classifier was trained, taking the outputs of the two-deep CNNs.

The authors in [42, 44, 57, 66, 69, 70, 72, 73, 90, 146, 147] applied pre-processing, augmentation, normalization, regularization, mixing FSM and FFDM MG images, and other techniques to better implement their network. Ge et al. [42] compared the performance of CNNs on pairs of FFDM and SFM obtained from the same patients with a time span of less than 3 months. Their results show that the CNN with FFDM images (AUC of 0.96) detect more MCs than the CNN with FSM images (AUC of 0.91). Hepsaug [74] achieved an accuracy of 88% when training separate deep CNN on only mass ROIs and 0.84% on training deep CNN on only MCs ROIs in the BCDR database. On the other hand, the accuracy results show that classifying only mass or only MCs is more successful compared to classifying mass and MCs data. Zhu et al. [20] conducted mass detection for whole MG images. Their deep multi-instance network uses linear regression with weight sharing for the malignant probability of each position from the CNN’s feature maps. The authors in [50, 146] trained a multi-stage CNN network for the classifications of lesions in MGs. Bekker et al. [56] presented a deep MV CNN for the classification of clustered breast MCs to two classes. Their results show that classification based on MV MGs show promising results. Carneiro et al. [32] addressed the classification of mass(es) using a pre-trained MV CNN. Their model classifies a full MG by extracting features from each view of the breast (train a separate CNN for each view) and combining these features in a joint CNN model to output a prediction that estimates the patient’s risk of developing breast cancer. Carneiro et al. [95] following his work [32] build a fully automated pre-trained CNN for detecting masses and MCs in MV MG images. Geras et al. [22] developed a MV CNN that utilizes large high-resolution images without downscaling. They showed that the accuracy of detecting and classifying MGs clearly increases with the size of the training data-set and that the best performance can only be achieved using the images in the original resolution. Yi et al. [23] utilized a deep MV learning by averaging the probability scores of both views to make the final prediction. Lotter et al. [65] introduced a multi-scale deep CNN trained with a curriculum learning strategy. Lotter et al. first train CNN-based patch classifiers on ROIs, and then use the learned features to initialize a scanning-based model that renders a decision on the whole image, having final results by averaging final scores across MV of the breast. Dhungel et al. [97, 98] presented an cascade DL networks for detecting, segmenting and classifying breast masses from MGs with minimal user intervention. Dhungel et al. [25], following their work in [52, 97, 98], implemented a MV deep residual neural network for the fully automated classification of MGs as either malignant or normal/benign (AUC of 0.8).

#### Risk assessment

The studies in [26, 27, 33, 34, 107, 115, 133] have demonstrated that applying CNNs methods have significant potential to develop a new short-term risk predicting scheme with improved performance in detecting early abnormal symptom from the negative MGs. Breast density is considered a strong indicator of breast cancer risk [26, 27, 33, 34, 152]. Fonseca et al. [26, 152] explored an automatic breast composition classification work-flow based on CNN for feature extraction in combination with a support vector machines classifier. Similar approach was done by Becker [33] achieving an (AUC of 0.82) comparable to experienced radiologists (AUC of 0.79–0.87).

Li [153] trained a deep CNN to estimate a probability map of breast density (PMD) to classify mammographic pixels into fatty class or dense class. Kallenberg et al. [27] presented an unsupervised CNN for breast density segmentation and automatic texture scoring. The model learns features across multiple scales, then they are fed to a simple classifier that is specific to the task of interest yielding AUC of 0.59. Ahn et al. [34] used CNN for the task of automatic classification of mammographic breast tissues into dense and fatty tissues. Their CNN is configured to learn the local features from image patches while keeping the context information of the whole MG. Wu et al. [107] managed to train a MV deep CNN using a data-set of 201,179 MGs for breast density classification. Mohamed et al. [115] achieved AUC of 0.95 when using only the MLO view images. In comparison, the AUC is 0.88 when using only the CC view images. When both the MLO and CC view images were combined as a single data-set, the AUC is lowered to 0.92. The authors in [110] following their work in [115] achieved better AUC of 0.98 by fine-tuning a pre-trained network. Hang [148] achieved classification accuracy of 66% for classification of full images into normal, benign and malignant.

#### Lesion localization

For localization, the information about which category an image belongs to is already available and the task is to instead figure out where exactly the object is located in the image. Classification and localization can also be combined so that a fixed amount of lesions in an image will be classified and also located. This task, called multi-class localization. The following authors employed CNNs in the aim of lesions classification and then localization within these images [14, 31, 45, 52–54, 57, 61, 98, 154, 155], potentially enabling E2E training. Ben-Ari et al. [24] introduced the detection of AD using a supervised pre-trained region-based network (R-CNN). Ertosun and Rubin [53] developed an E2E dual CNN based visual search system for localization of mass(es) in MGs. Kisilev et al. [61] gave a semantic description for MGs. The authors presented a multi-task R-CNN approach for detection and semantic description of lesions in diagnostic images. Carneiro and Bradley [54] presented an automated supervised architecture composes of a multi-scale deep belief network that selects suspicious regions to be further processed by a two-level cascaded R-CNN. Akselrod et al. [45] integrated several cascaded segmentation modules into a modified cascaded R-CNN. Hwang et al. [51] proposed a self-transfer learning framework which enables training CNNs for object localization without neither any location information nor pre-trained models. Zhu et al. [57] introduced an E2E adversarial training for mammographic mass segmentation to learn robustly from scarce MGs. The authors highlighted the importance of pre-processing, augmentation, image enhancement, and normalization techniques. The authors stated that it is not feasible to use networks pre-trained on general images since ROI characteristics of medical images are thoroughly different from natural images. However, their opinion contradicts other researchers work.

The authors in [31, 52, 155] proposed a patch-based CNN to detect masses. Choukroun et al. [155] proposed a method that classifies MGs by detecting discriminative local information contained in patches through a deep CNN and then uses the local information to localize tumors. Dhungel et al. [52] used the output from a CNN as a complimentary potential function to a deep belief network (DBN) models for the localization of breast masses from MGs, using a small training data-set. A drawback of the patch-based approach in [31, 52] is that the input patches came from non-overlapping areas, which makes it difficult to preciously localize masses. Moreover, the size of the input patches in [31, 52] is very small that produces a difficulty in differentiating normal tissues from abnormal ones.

The authors in [14, 154] used the famous YOLO-based deep CNN [83] for breast mass classification and localization. The trained YOLO-based system localizes the masses and classifies their types into benign or malignant. The authors in [154] achieved a mass location with an overall accuracy of 96.33% and detection of benign and malignant lesions with an overall accuracy of 85.52%.

#### Image retrieval

Tasks like medical image retrieval using DL have been lately addressed in the medical field to facilitate the process of production and management of large medical image databases. Conventional methods for analyzing medical images have achieved limited success, as they are not capable to tackle the huge databases. The learned features and the classification results from training a CNN are used to retrieve medical images. Qayyum et al. [114] proposed a DL based framework for content based medical image retrieval (CBMIR) by training a deep CNN for the classification tasks using medical images for different body organs (e.g. MGs, lungs, brain, liver etc. Qayyum et al. [114] achieved an average classification accuracy of 99.77% for 24 classes of medical images. Similarly, Ahmad et al. [156] trained a deep CNN for CBMIR of different 193 classes for different body organs. Moreover, [156] applied TL and augmentation to increase the performance of their deep CNN.

#### Super resolution image reconstruction

The task of super resolution image reconstruction using CNN (SRCNN) is an E2E mapping between the low and high-resolution images for enhancing images [157]. The mapping is represented as a deep CNN that takes the low resolution image as the input and outputs the high resolution one. The study of Umehara et al. [158] shows that SRCNN can significantly outperform conventional interpolation methods for enhancing image resolution in digital mammography especially in dense breasts.

### Research challenges and directions

In this section, we list the research challenges and directions that require further investigations by the community.

#### Localization of tumors

The patch-based CNNs, R-CNNs, Fast R-CNNs, Faster R-CNNs, and YOLO methods have recently become more popular for localization tasks for MGs. Faster R-CNN is the choice of most of the mammography researchers who aim to obtain high detection accuracy numbers. However, training a R-CNN and its variants faster versions is time-consuming and memory expensive. In contrast, for faster computations, less accurate detection, and limited memory computations, the YOLO method is the right choice. Finally, patch-based CNN methods are not recommended and result in many false positives. More research need to be done for better localization of tumors in MGs.

#### Limited data for learning

One of the challenging problems that face researchers while training CNNs is the size of the training data-set. As discussed in the best practice section, although several approaches such as data augmentation, TL, and drop out have been used to handle the problem of training the model with limited samples, this problem has remained challenging.

#### Imbalanced data-set

Another challenging problem is the imbalance ratio between positive and negative classes in the training data-sets. Training CNN models directly on imbalanced data-sets may bias the prediction towards the more common classes like normal. The effect of imbalanced data-set on the performance of a CNN for MGs has not been studied thoroughly. Some works used balanced data-set and some used imbalanced ones. Since in general less abnormal MGs are available compare to normal MGs it is very important to investigate the effect of using balanced and imbalanced data-sets on the accuracy of the CNN model.

#### Size of lesions

The size variation of lesions within MG images is another challenge for training CNNs in detecting cancer. Resizing a large MG to 224 ×224 or 227 ×227 (common choices among researchers) will likely make the ROI hard to detect and/or classify. To address this problem, several studies have proposed to train a CNN model using different scales of lesions [27, 54, 65]. More research is required to find lesions of different sizes.

#### Memory constrains

The classification of whole size MG images is challenging due to the memory constraints and increased feature space. Researchers in [22, 128] address this problem by resizing the images to smaller ones, however, this affects the accuracy of their model. More research should be done on how to overcome the memory constraints while training CNNs with full-size MG images.

#### Non-annotated data-set

Another challenging problem to researchers is how to train a CNN model using a non-annotated data-set. In non-annotated data-set, the input image to CNN model is binary labeled as normal or cancerous without any details about the location of the abnormalities. To address this problem, Lotter at el. [65] train a patch-level CNN classifier, which is then used as feature extractor to an image-level model. Training the CNNs for classification of non-annotated data-set is still an open area for research [20, 65, 129].

#### False positives reduction

Even though CNNs are very successful in providing better performance compared to traditional CADs, they still result in false positives. False positive results cause patients needless anxiety, additional testing, biopsies, and unnecessary costs. Several approaches have been proposed to improve false positive in CNNs such as using MV CNNs [15, 20–25, 95, 107, 108]. However, more research is required to integrate prior images with current screening to eliminate false positives.

#### Multiple detection

Current CNN models are trained to detect and/or localize mass(es) within MGs neglecting the existence of MCs. More research should be directed on detecting multiple abnormalities within the same breast.

#### Pre-processing filters

In FSM images, a significant number of abnormalities are misdiagnosed or missed due to the less visibility, low contrast, poor quality, and noisy nature of these images. Common pre-processing techniques (e.g. CLAHE, median filter) are proposed in [62, 89–91] to enhance image quality, image smoothing and noise reduction. However, choosing the proper pre-processing technique for MGs in order to improve the classification of CNNs is still an open problem.

## Discussion and recommendations

**Fig. 3:**
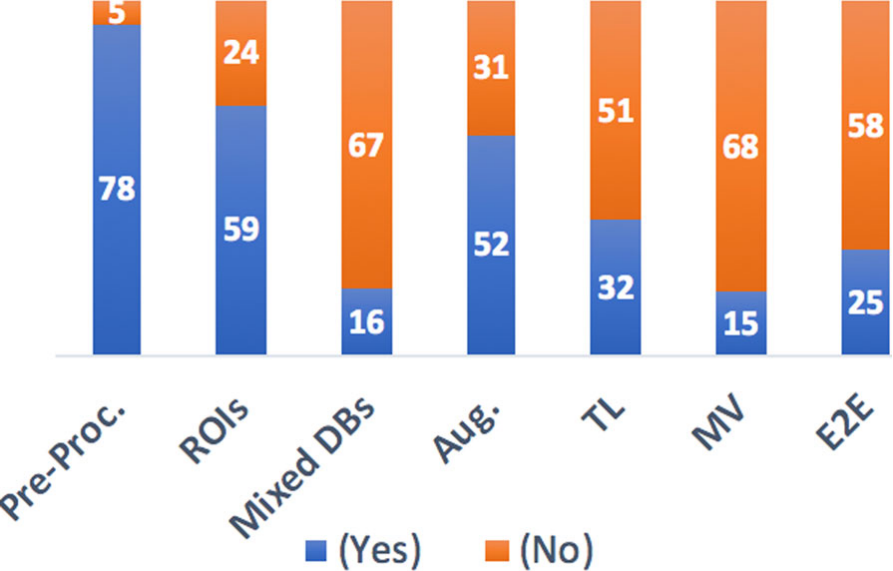
Statistics for the included studies
- Apply cropping and down sampling for more efficient computation.
- Use a suitable validation approach according to the size of the data-set available.
- Use augmentation, drop-out, and TL to reduce overfitting and increase the generalization of the model.
- Use suitable batch size if using ROIs.
- Use multi-view (MV) CNNs to embed more information for better performance.
- Use full resolution images if it is computationally practical.
- Mix between FFDM and FSM images.
- Use suitable activation function such as ReLU, be careful with initializing the learning rates and possibly monitor the fraction of dead neurons in the network.
- Use large well labeled data-set if available.
- Go deeper in layers if large data-set is available.
- Use context and patient information in multi-modal models.
- Use recently available libraries for implementing CNNs such as Tensorflow or Keras.

We show a breakdown of the studies included in this survey grouped by their neural network task (see Additional file 1: Table S1). Figure 3, shows the percentage of studies employing some of the CNN best practices that are discussed in the previous section and are shown in Additional file 1: Table S1. 78 studies (out of 83) used common pre-processing techniques to enhance the quality of images, reduce or remove noise, and improve the contrast of MGs. That shows the importance of having a good separation between foreground and background pixels and not removing the important information from the images. Moreover, 59 studies used ROIs for more efficient computation, while 23 studies applied CNN to MG of full image size as in [20, 22, 26, 32, 45, 51, 57, 62]. Even for CNNs that are trained with full image size, the pre-processing is mandatory to remove marks, labels, pectoral muscle and black areas that can interfere in the post-processing of these images. Data augmentation has been recommended and employed by 52 studies. Data augmentation reduces overfitting by generating more instances of training data. TL is gaining more popularity for medical images, 32 studies have successfully applied it to pre-train their network. From 2015 until now, there is an increasing trend in using TL. 15 studies implemented a MV CNNs which lead to significant improvements in the performance of the single-view ones. It is a beneficial practice to use both CC and MLO views to detect abnormalities. 25 studies implemented an E2E CNN which may include segmentation, detection, and classification of lesions in MGs. We summarize the recommendations to significantly improve the performance of CNNs in detection and classification of breast cancer using MG images as follows:

- Use pre-processing techniques such as CLAHE filter to improve the contrast of MGs, median filter to reduce noise, and un-sharp masking to smooth the images.

## Conclusions

In this survey, we conducted a detailed review of the strengths, limitations, and performance of the most recent CNNs applications in analyzing mammogram (MG) images. This survey systematically compares recent approaches of CNNs in MG images, and show how the advances in DL methods give promising results that can aid radiologists and serve as a second eye for them. The potential role of CNN methods is to handle millions of routine imaging exams, presenting the potential cancers to the radiologists who perform follow-up procedures. We discuss the currently publicly available MG databases. We also give a deep insight into the architectures of CNNs used for various tasks in mammography.

This survey represents a valuable resource for the mammography research community since it can be utilized as a basis in their current and future studies. The given comparison among common publicly available MG repositories guides the community to select the most appropriate database for their application(s). Moreover, this survey lists the best practices that improve the performance of CNNs including the pre-processing of images and the use of multi-view images. In addition, other listed techniques like transfer learning (TL), data augmentation, batch normalization, and dropout are appealing solutions to reduce overfitting and increase the generalization of the CNN model. Finally, we identified research challenges and directions that require further investigations for mammography.

## Additional file

Additional file 1 Supplementary Table 1, a comparison between different approaches in literature. (PDF 98 kb)

## Abbreviations

ACC: Accuracy
AUC: Area under the receiver operating curve
AD: Architectural distortion
BACs: Breast arterial calcifications
BCDR: Breast cancer digital repository
BN: Batch normalization
CAD: Computer-aided detection
CBMIR: Content based medical image retrieval
CC: Craniocaudal
CRF: Conditional random field
CLAHE: Contrast limited adaptive histogram equalization
CNNs: Deep convolutional neural networks
DBT: Digital breast tomosynthesis
DDMS: Digital database for screening mammography
DL: Deep learning
E2E: End-to-end
FC: Fully connected layer
FFDM: Digital mammography
FN: False negative
FP: False positive
FPR: False positive rate
FSMs: Film screen mammograms
HGD: Histogram of the gradient divergence
HOG: Histogram of oriented gradients
IRMA: Image retrieval in medical applications
MCs: Calcifications
MG: Mammogram
MIAS: Mammographic Image Analysis Society
ML: Machine learning
MLO: Mediolateral-oblique
MLP: Multilayer perceptron
PMD: Probability map of breast density
Pool: Pooling layer
R-CNN: Region-based network
ReLU: Rectified liner unit
ROC: Receiver operating characteristic curve
ROIs: Region of interests
SRCNN: Super resolution image reconstruction
SSL: Semi-supervised learning
SVM: Support vector machine
TL: Transfer learning
TN: True negative
TP: True positive
TPR: True positive rate
