# Supplementary material for: Deep convolutional neural networks for mammography: advances, challenges and applications
Source: BMC Bioinformatics. 2019 Jun 6;20(Suppl 11):281. doi: 10.1186/s12859-019-2823-4 (PMC6551243; doi:10.1186/s12859-019-2823-4)
Supplement: Supplementary file 1 — Supplementary Table 1, a comparison between different approaches in literature. (PDF 98 kb) [file 12859_2019_2823_MOESM1_ESM.pdf]

Supplementary Table 1: A comparison between different approaches in literature from the point of view 1) author, 2) database used, 3) number of images used before and after augmentation in parenthesis, 4) the use of full image or ROI, 5) if pre-processing is done, 6) size of images used in pixel, 7) if augmentation is applied, 8) if it is end-to-end (E2E) technique, 9) if transfer learning (TL) is used, 10) muli-view (MV) or single-view (SV), 11) area under curve (AUC), 12) accuracy (Acc.%), 13) class used: normal images (NL); benign images (B); malignant (M); calcifications (MCs); architectural distortion (AD), 14) lesion being segmented, 15) number of layers in the architecture.

**Author**	**Year**	**Database**	**#Images**	**Kind**	**Pre-pro.**	**Size**	**AUG.**	**E2E**	**TL**	**View**	**AUC**	**Acc.%**	**Class**	**Lesion**	**Layers**
							**Lesion classification**								
Sahiner [1]	1996	Private	168(672)	ROIs	Y	32×32	Y	N	N	SV	0.87	-	NL-Mass	Mass	3
Lo [2]	2002	MIAS	200(3,193)	ROIs	Y	512×512	Y	N	N	SV	0.89	-	NL-Mass	Mass	3
Domingues [3]	2013	INbreast	112	ROIs	N	32×32	N	N	N	SV	0.89	-	NL-Mass	Mass	3
Dhungel [4]	2015	BCRP, INbreast	116-77	ROIs	N	40×40	N	N	N	SV	0.9	-	NL-Mass	Mass	6
Kooi [5]	2016	Private	398(2.5M)	ROIs	Y	250×250	Y	N	N	SV	0.87		NL-Mass	Mass	11
Wichakam [6]	2016	INbreast	216(1,728)	ROIs	Y	32×32	Y	N	N	SV	-	98.4	NL-Mass	Mass	9
Zhu [7]	2017	INbreast	410	Image	Y	224×224	Y	Y	Y	MV	0.85	90	NL-Mass	Mass	8
Suzuki [8]	2016	DDSM	1,656	ROIs	Y	454×454	N	N	Y	SV	-	-	NL-Mass	Mass	8
Dubrovina [9]	2016	N/A	40(8k)	ROIs	Y	256×256	Y	N	N	SV	-	-	NL-Mass	Mass	7
Suzuki [10]	2016	DDSM	1,656	ROIs	Y	454×454	N	N	Y	SV	-	-	NL-Mass	Mass	8
Kooi [11]	2017	Private	398(2.5M)	ROIs	Y	250×250	Y	N	Y	MV	0.87	-	NL-Mass	Mass	22
Sun [12]	2017	Private	3158(25K)	ROIs	Y	52×52	Y	N	N	SV	0.88	82.2	NL-Mass	Mass	7
Samala [13]	2016	Private	2461(45072)	ROI	Y	128×128	Y	N	N	SV	0.99	-	NL-Mass	Mass	8
Hadad [14]	2017	Private	11092(32,064)	ROI	Y	32×32	Y	N	N	SV	0.94	87	NL-Mass	Mass	13
Chan [15]	1995	Private	52(1,744)	ROIs	Y	16×16	Y	N	N	SV	0.91	-	NL-MCs	MCs	3
Gardezi [16]	2017	IRMA	2795(25k)	ROIs, Image	Y	224×224	N	N	N	SV	1	100	NL-Mass	Mass	16
Ge [17]	2007	Private	192	ROIs	Y	16×16	Y	N	N	SV	0.96	-	NL-MCs	MCs	3
Valvano [18]	2017	Private	290(90,000)	ROIs	Y	99×99	Y	N	N	SV	-	83.7	NL-MCs	MCs	10
Mordang [19]	2016	Private	1606(45M)	ROIs	Y	13×13	Y	N	N	MV	-	-	NL-MCs	MCs	9
Wang [20]	2017	Private	840(1.94M)	ROIs	Y	95×95	Y	N	N	MV	-	-	NL-MCs	MCs	12
Bria [21]	2017	Private	7,579(≈27M)	ROIs	Y	12×12	Y	N	N	SV	-	-	NL-MCs	MCs	8
Ben-Ari [22]	2017	DDSM	136(21k)	ROIs	Y	224×224	Y	N	Y	MV	-	80	NL-AD	AD	9
Jamieson [23]	2012	Private	739(2,393)	ROIs	Y	140×140	Y	N	N	SV	0.71	-	B-M	Mass	4
Arevalo [24]	2015	INbreast	736(5,152)	ROIs	Y	150×150	Y	Y	Y	SV	0.82	-	B-M	Mass	7
Agarwal [25]	2015	DDSM	8752(50k)	ROIs	Y	64×64	Y	N	N	SV	-	90	B-M	Mass, MCs	8
Jiao [26]	2016	DDSM	600(1,800)	ROIs	Y	227×227	Y	N	Y	SV	-	96.7	B-M	Mass	9
Bekker [27]	2016	DDSM	1410	ROIs	N	N/A	N	N	N	SV	0.89	78.7	B-M	MCs	3
Sharma [28]	2016	DDSM	40	ROIs	Y	N/A	N	N	N	SV	0.85	79.2	B-M	Mass	N/A
Levy [29]	2016	DDSM	1820(36k)	ROIs	Y	224×224	Y	Y	N	SV	-	92.9	B-M	Mass	9
Abbas [30]	2016	MIAS, DDSM	600	ROIs	Y	250×250	N	Y	N	SV	0.91	91.5	B-M	Mass	N/A
Huynh [31]	2016	Private	607	ROIs	Y	256×256	N	N	Y	SV	0.86	-	B-M	Mass	8

Continuation of Table 1

Author	Year	Database	#Images	Kind	Pre-pro.	Size	AUG.	E2E	TL	View	AUC	Acc.%	Class	Lesion	Layers
**Lesion classification**															
Arevalo [32]	2016	BCDR	736(5k)	ROIs	Y	150×150	Y	N	Y	SV	0.86	-	B-M	Mass	7
Jiang [33]	2017	BCDR	736(N/A)	ROIs	Y	227×227	Y	N	Y	SV	0.88	-	B-M	Mass	22
Samala [34]	2017	DDSM, Private	322(2576) 8334(17056)	ROI	Y	128×128	Y	N	Y	SV	0.82	-	B-M	Mass	10
Sert [35]	2017	DDSM	2620(5965)	ROIs	Y	224×224	Y	N	Y	SV	-	94.3	B-M	MCs	22
Jiao [36]	2017	DDSM	600(1,800)	ROIs	Y	227×227	Y	N	Y	SV	-	92.5	B-M	Mass	8
Jaffar [37]	2017	MIAS, DDSM.	2122(19k)	ROIs	Y	96×96	N	N	N	SV	0.93	93.4	B-M	Mass	8
Chougrad [38]	2017	BCDR	600(N/A)	ROIs	Y	299×299	Y	Y	Y	SV	0.96	97.5	B-M	Mass	221
Bakkouri [39]	2017	DDSM, BCDR.	10k(60k)	ROIs	Y	32×32	Y	N	N	SV	-	97.3	B-M	Mass	7
Antropova [40]	2017	Private	739	ROIs	Y	512×512	N	N	Y	SV	0.86	-	B-M	Mass	19
Qiu [41]	2017	Private	560	ROIs	Y	64×64	N	N	N	SV	0.79	-	B-M	Mass	8
Gallego [42]	2016	MIAS	322(600)	ROIs	Y	227×227	Y	N	Y	SV	-	64.5	NL-B-M	Mass	8
Jadoon [43]	2017	IRMA	2796 (19k)	ROIs	Y	28×28	Y	N	N	SV	-	83.7	NL-B-M	Mass	5
Yi [44]	2017	DDSM	2085(N/A)	ROIs	Y	224×224	Y	N	Y	MV	0.91	85	NL-B-M	Mass	22
Teare [45]	2017	DDSM, ZMDS	>6000(N/A) 1739(N/A)	Image	Y	299×299	Y	N	Y	SV	0.92	-	NL-B-M	Mass, MCs	22
Hepsaug [46]	2017	MIAS, BCDR	1160-2474	ROIs	Y	50×50	N	N	N	SV	-	68, 62	NL-B-M	Mass, MCs	N/A
Hang [47]	2017	DDSM	1318	Image	Y	521×521	N	N	N	MV	-	66	NL-B-M	Mass	13
Kooi [48]	2017	Private	490	ROIs	Y	250×250	N	N	N	SV	0.87	-	Mass-MCs	Mass, MCs	N/A
Qiu [49]	2016	N/A	560	ROIs	Y	512×512	N	Y	N	SV	0.8	-	B-M	Mass	8
Dhungel [50]	2016	INbreast	116(1160)	ROIs	Y	40×40	Y	Y	Y	SV	-	84	B-M	Mass	5
Geras [51]	2017	Private	102,800	Image	Y	$2k \times 2k$	N	Y	N	MV	0.76	-	NL-M	Mass, MCs	11
Kooi [52]	2017	Private	44K(1.3M)	ROIs	Y	250×250	Y	Y	N	SV	0.94	-	NL-M, B-M	Mass	7
Dhungel [53]	2017	INbreast	512	Image	Y	120×120	Y	Y	Y	MV	0.8	-	NL-B-M	Mass, MCs	392
Lotter [54]	2017	DDSM	10480(N/A)	Image	Y	256×256	Y	Y	Y	MV	0.92	-	NL-B-M	Mass, MCs	152
Ribli [55]	2017	DDSM, INbreast	26020(N/A), 115	Image	Y	$2.1k\times1.7k$	Y	Y	N	SV	0.95	-	NL-B-M	Mass, MCs	16
Akselrod [56]	2017	Private	860	Image	Y	224×224	N	Y	Y	SV	0.72	77	NL-B-M	Mass	16
Carneiro [57]	2015	DDSM, INbreast	680-410	Image	Y	264×264	N	Y	Y	MV	0.97	-	B-M	Mass	8
Carneiro [58]	2017	DDSM, INbreast	680-410	Image	Y	264×264	N	N	Y	MV	0.99-0.91	-	NL-B-M	Mass, MCs	8
Kooi [59]	2017	Private	201,851(N/A)	ROIs	Y	250×250	Y	N	N	MV	0.88	-	NL-Mass	Mass	19
**Lesion localization**															
Ertosun [60]	2015	DDSM	2420(1.8M)	ROIs	Y	256×256	Y	Y	Y	SV	-	85	NL-Mass	Mass	22
Hwang [61]	2016	MIAS, DDSM	15,837	Image	Y	500×500	N	N	N	SV	0.89	84.1	NL-Mass	Mass	7

Continuation of Table 1															
Author	Year	Database	#Images	Kind	Pre-pro.	Size	AUG.	E2E	TL	View	AUC	Acc.%	Class	Lesion	Layers
**Lesion localization**															
Akselrod [62]	2016	Private	850(4,75)	Image	N	800×800	Y	Y	N	SV	-	-	NL-B-M	Mass, MCs	16
Carneiro [63]	2016	BCRP, INbreast	316(3,160), 410(4.1k)	ROIs	Y	40×40	Y	Y	N	SV	-	-	NL-B-M	Mass	8
Choukroun [64]	2017	Private	2,500(28k)	ROIs	Y	224×224	Y	N	Y	SV	0.83	-	NL-B-M	Mass, MCs	8
Zhu [65]	2016	BCRP, INbreast	171(680), 116(464)	ROIs	Y	40×40	Y	Y	N	SV	-	91.3	NL-M	Mass	4
Dhungel [66]	2017	INbreast	410(4.1k)	ROIs	Y	40×40	Y	Y	Y	SV	0.76	91	B-M	Mass	5
Kisilev [67]	2016	DDSM	512	ROIs	Y	128×128	N	N	N	SV	-	-	B-M	Mass	7
Al-masni [68]	2017	DDSM	600	Image	Y	448×448	N	N	Y	SV	0.87	85.5	B-M	Mass	27
Platania [69]	2017	IRMA	10,480(25k)	ROIs, Image	Y	128×128	Y	Y	Y	SV	0.92	93.5	B-M	Mass	19
Sun [70]	2016	Private	420(42k)	ROIs	Y	52×52	N	N	N	MV	0.72	-	B-M	Mass	8
**Risk assessment**															
Qiu [71]	2016	Private	270	ROIs	Y	256×256	N	N	N	SV	-	71.4	BI-RADS	Density	8
Fonseca [72]	2015	Private	729	Image	N	200×200	N	N	N	SV	-	73	BI-RADS	Density	3
Fonseca [73]	2016	Private	1060(N/A)	Image	Y	200×200	Y	N	Y	SV	-	-	BI-RADS	Density	3
Becker [74]	2017	BCDR	286(N/A)	Image	Y	N/A	Y	Y	N	SV	0.81	-	BI-RADS	Density	N/A
Kallenberg [75]	2016	Private	1,555	ROIs	Y	24×24	N	N	N	SV	-	59	BI-RADS	Density	6
Ahn [76]	2017	Private	10,94(N/A)	ROIs	Y	41×41	Y	N	N	SV	-	-	BI-RADS	Density	16
Li [77]	2017	Private	661(1M)	ROIs	Y	61×61	Y	N	N	SV	-	-	BI-RADS	Density	6
Wu [78]	2017	Private	201,179(N/A)	Image	Y	2.6k×2k	Y	Y	Y	MV	0.93	-	BI-RADS	Density	19
Thomaz [79]	2017	Private	307	Image	Y	260×200	N	N	N	SV	-	98.4	BI-RADS	Density	N/A
Mohamed [80]	2017	Private	15,415	Image	Y	227×227	N	N	N	SV	0.92	-	BI-RADS	Density	8
Mohamed [81]	2017	Private	6000	Image	Y	227×227	N	N	Y	SV	0.98	-	BI-RADS	Density	8
**Image retrieval**															
Qayyum [82]	2017	Multiple	7200	Image	Y	224×224	N	Y	N	SV	-	99.8	24 classes	All	8
Ahmad [83]	2017	IRMA	15363(68k)	ROIs	Y	224×224	Y	Y	Y	SV	0.75	-	193 classes	All	16
**Resolution image reconstruction**															
Umehara [84]	2017	CBIS-DDSM	711	Image	Y	variable	Y	Y	N	SV	-	-	Same image	Any	23

References

1. Sahiner B, Chan HP, Petrick N, Wei D, Helvie MA, Adler DD, et al. Classification of mass and normal breast tissue: a convolution neural network classifier with spatial domain and texture images. IEEE transactions on Medical Imaging. 1996;15(5):598–610.
2. Lo SCB, Li H, Wang Y, Kinnard L, Freedman MT. A multiple circular path convolution neural network system for detection of mammographic masses. IEEE transactions on medical imaging. 2002;21(2):150–158.
3. Domingues I, Cardoso J. Mass detection on mammogram images: a first assessment of deep learning techniques. In: 19th Portuguese Conference on Pattern Recognition (RECPAD); 2013. .
4. Dhungel N, Carneiro G, Bradley AP. Deep learning and structured prediction for the segmentation of mass in mammograms. In: International Conference on Medical Image Computing and Computer-Assisted Intervention. Springer; 2015. p. 605–612.
5. Kooi T, Gubern-Merida A, Mordang JJ, Mann R, Pijnappel R, Schuur K, et al. A comparison between a deep convolutional neural network and radiologists for classifying regions of interest in mammography. In: International Workshop on Digital Mammography. Springer; 2016. p. 51–56.
6. Wichakam I, Vateekul P. Combining deep convolutional networks and SVMs for mass detection on digital mammograms. In: Knowledge and Smart Technology (KST), 2016 8th International Conference on. IEEE; 2016. p. 239–244.
7. Zhu W, Lou Q, Vang YS, Xie X. Deep multi-instance networks with sparse label assignment for whole mammogram classification. In: International Conference on Medical Image Computing and Computer-Assisted Intervention. Springer; 2017. p. 603–611.
8. Suzuki S, Zhang X, Homma N, Ichiji K, Sugita N, Kawasumi Y, et al. Mass detection using deep convolutional neural network for mammographic computer-aided diagnosis. In: Society of Instrument and Control Engineers of Japan (SICE), 2016 55th Annual Conference of the. IEEE; 2016. p. 1382–1386.
9. Dubrovina A, Kisilev P, Ginsburg B, Hashoul S, Kimmel R. Computational mammography using deep neural networks. Computer Methods in Biomechanics and Biomedical Engineering: Imaging & Visualization. 2016;p. 1–5.
10. Suzuki S, Zhang X, Homma N, Ichiji K, Kawasumi Y, Ishibashi T, et al. WE-DE-207B-02: Detection of Masses On Mammograms Using Deep Convolutional Neural Network: A Feasibility Study. Medical physics. 2016;43(6):3817–3817.
11. Kooi T, Ginneken B, Karssemeijer N, Heeten A. Discriminating solitary cysts from soft tissue lesions in mammography using a pretrained deep convolutional neural network. Medical physics. 2017;44(3):1017–1027.
12. Sun W, Tseng TLB, Zhang J, Qian W. Enhancing deep convolutional neural network scheme for breast cancer diagnosis with unlabeled data. Computerized Medical Imaging and Graphics. 2017;57:4–9.
13. Samala RK, Chan HP, Hadjiiski L, Helvie MA, Wei J, Cha K. Mass detection in digital breast tomosynthesis: Deep convolutional neural network with transfer learning from mammography. Medical physics. 2016;43(12):6654–6666.
14. Hadad O, Bakalo R, Ben-Ari R, Hashoul S, Amit G. Classification of breast lesions using cross-modal deep learning. In: Biomedical Imaging (ISBI 2017), 2017 IEEE 14th International Symposium on. IEEE; 2017. p. 109–112.
15. Chan HP, Lo SCB, Sahiner B, Lam KL, Helvie MA. Computer-aided detection of mammographic microcalcifications: Pattern recognition with an artificial neural network. Medical Physics. 1995;22(10):1555–1567.
16. Gardezi SJS, Awais M, Faye I, Meriaudeau F. Mammogram classification using Deep learning. 2017;.
17. Ge J, Hadjiiski LM, Sahiner B, Wei J, Helvie MA, Zhou C, et al. Computer-aided detection system for clustered microcalcifications: comparison of performance on full-field digital mammograms and digitized screen-film mammograms. Physics in medicine and biology. 2007;52(4):981.
18. Valvano G, Della Latta D, Martini N, Santini G, Gori A, Iacconi C, et al. Evaluation of a Deep Convolutional Neural Network method for the segmentation of breast microcalcifications in Mammography Imaging. In: EMBEC & NBC 2017. Springer; 2017. p. 438–441.
19. Mordang JJ, Janssen T, Bria A, Kooi T, Gubern-Mérida A, Karssemeijer N. Automatic microcalcification detection in multi-vendor mammography using convolutional neural networks. In: International Workshop on Digital Mammography. Springer; 2016. p. 35–42.
20. Wang J, Ding H, Azamian F, Zhou B, Iribarren C, Molloi S, et al. Detecting cardiovascular disease from mammograms with deep learning. IEEE transactions on medical imaging. 2017;.
21. Bria A, Marrocco C, Galdran A, Campilho A, Marchesi A, Mordang JJ, et al. Spatial Enhancement by Dehazing for Detection of Microcalcifications with Convolutional Nets. In: International Conference on Image Analysis and Processing. Springer; 2017. p. 288–298.
22. Ben-Ari R, Akselrod-Ballin A, Karlinsky L, Hashoul S. Domain specific convolutional neural nets for detection of architectural distortion in mammograms. In: Biomedical Imaging (ISBI 2017), 2017 IEEE 14th International Symposium on. IEEE; 2017. p. 552–556.
23. Jamieson AR, Drukker K, Giger ML. Breast image feature learning with adaptive deconvolutional networks. SPIE Medical Imaging, Strony. 2012;2012:831506–831506.
24. Arevalo J, González FA, Ramos-Pollán R, Oliveira JL, Lopez MAG. Convolutional neural networks for mammography mass lesion classification. In: Engineering in Medicine and Biology Society (EMBC), 2015 37th Annual International Conference of the IEEE. IEEE; 2015. p. 797–800.
25. Agarwal V, Carson C. Using Deep Convolutional Neural Networks to predict semantic features of lesions in mammograms. C231n Course Project Reports. 2015;.
26. Jiao Z, Gao X, Wang Y, Li J. A deep feature based framework for breast masses classification. Neurocomputing. 2016;197:221–231.
27. Bekker AJ, Greenspan H, Goldberger J. A multi-view deep learning architecture for classification of breast microcalcifications. In: Biomedical Imaging (ISBI), 2016 IEEE 13th International Symposium on. IEEE; 2016. p. 726–730.
28. Sharma K, Preet B. Classification of mammogram images by using CNN classifier. In: Advances in Computing, Communications and Informatics (ICACCI), 2016 International Conference on. IEEE; 2016. p. 2743–2749.
29. Lévy D, Jain A. Breast mass classification from mammograms using deep convolutional neural networks. arXiv preprint arXiv:161200542. 2016;.
30. Abbas Q. DeepCAD: A Computer-Aided Diagnosis System for Mammographic Masses Using Deep Invariant Features. Computers. 2016;5(4):28.
31. Huynh BQ, Li H, Giger ML. Digital mammographic tumor classification using transfer learning from deep convolutional neural networks. Journal of Medical Imaging. 2016;3(3):034501–034501.
32. Arevalo J, González FA, Ramos-Pollán R, Oliveira JL, Lopez MAG. Representation learning for mammography mass lesion classification with convolutional neural networks. Computer methods and programs in biomedicine. 2016;127:248–257.
33. Jiang F, Liu H, Yu S, Xie Y. Breast mass lesion classification in mammograms by transfer learning. In: Proceedings of the 5th International Conference on Bioinformatics and Computational Biology. ACM; 2017. p. 59–62.
34. Samala RK, Chan HP, Hadjiiski LM, Helvie MA, Cha KH, Richter CD. Multi-task transfer learning deep convolutional neural network: application to computer-aided diagnosis of breast cancer on mammograms. Physics in Medicine & Biology. 2017;62(23):8894.
35. Sert E, Ertekin S, Halici U. Ensemble of convolutional neural networks for classification of breast microcalcification from mammograms. In: Engineering in Medicine and Biology Society (EMBC), 2017 39th Annual International Conference of the IEEE. IEEE; 2017. p. 689–692.
36. Jiao Z, Gao X, Wang Y, Li J. A parasitic metric learning net for breast mass classification based on mammography. Pattern Recognition. 2017;.
37. Jaffar MA. Deep Learning based Computer Aided Diagnosis System for Breast Mammograms. INTERNATIONAL JOURNAL OF ADVANCED COMPUTER SCIENCE AND APPLICATIONS. 2017;8(7):286–290.
38. Chougrad H, Zouaki H, Alheyane O. Convolutional Neural Networks for Breast Cancer Screening: Transfer Learning with Exponential Decay. arXiv preprint arXiv:171110752. 2017;.

39. Bakkouri I, Afdel K. Breast tumor classification based on deep convolutional neural networks. In: Advanced Technologies for Signal and Image Processing (ATSIP), 2017 International Conference on. IEEE; 2017. p. 1–6.
40. Antropova N, Huynh BQ, Giger ML. A Deep Feature Fusion Methodology for Breast Cancer Diagnosis Demonstrated on Three Imaging Modality Datasets. Medical physics. 2017;.
41. Qiu Y, Yan S, Gundreddy RR, Wang Y, Cheng S, Liu H, et al. A New Approach to Develop Computer-Aided Diagnosis Scheme of Breast Mass Classification Using Deep Learning Technology. Journal of X-Ray Science and Technology. 2017;(Preprint):1–13.
42. Gallego-Posada J, Montoya-Zapata D, Quintero-Montoya O. Detection and Diagnosis of Breast Tumors using Deep Convolutional Neural Networks;.
43. Jadoon MM, Zhang Q, Haq IU, Butt S, Jadoon A. Three-Class Mammogram Classification Based on Descriptive CNN Features. BioMed research international. 2017;2017.
44. Yi D, Sawyer RL, Cohn III D, Dunnmon J, Lam C, Xiao X, et al. Optimizing and Visualizing Deep Learning for Benign/Malignant Classification in Breast Tumors. arXiv preprint arXiv:170506362. 2017;.
45. Teare P, Fishman M, Benzaquen O, Toledano E, Elnekave E. Malignancy Detection on Mammography Using Dual Deep Convolutional Neural Networks and Genetically Discovered False Color Input Enhancement. Journal of Digital Imaging. 2017;30(4):499–505.
46. Hepsağ PU, Özel SA, Yazıcı A. Using deep learning for mammography classification. In: Computer Science and Engineering (UBMK), 2017 International Conference on. IEEE; 2017. p. 418–423.
47. Hang W, Liu Z, Hannun A. GlimpseNet: Attentional Methods for Full-Image Mammogram Diagnosis;.
48. Kooi T, Mordang JJ, Karssemeijer N. Conditional Random Field Modelling of Interactions Between Findings in Mammography. In: SPIE Medical Imaging. International Society for Optics and Photonics; 2017. p. 101341E–101341E.
49. Qiu Y, Yan S, Tan M, Cheng S, Liu H, Zheng B. Computer-aided classification of mammographic masses using the deep learning technology: a preliminary study. In: SPIE Medical Imaging. International Society for Optics and Photonics; 2016. p. 978520–978520.
50. Dhungel N, Carneiro G, Bradley AP. The automated learning of deep features for breast mass classification from mammograms. In: International Conference on Medical Image Computing and Computer-Assisted Intervention. Springer; 2016. p. 106–114.
51. Geras KJ, Wolfson S, Kim S, Moy L, Cho K. High-Resolution Breast Cancer Screening with Multi-View Deep Convolutional Neural Networks. arXiv preprint arXiv:170307047. 2017;.
52. Kooi T, Litjens G, van Ginneken B, Gubern-Mérida A, Sánchez CI, Mann R, et al. Large scale deep learning for computer aided detection of mammographic lesions. Medical image analysis. 2017;35:303–312.
53. Dhungel N, Carneiro G, Bradley AP. Fully automated classification of mammograms using deep residual neural networks. In: Biomedical Imaging (ISBI 2017), 2017 IEEE 14th International Symposium on. IEEE; 2017. p. 310–314.
54. Lotter W, Sorensen G, Cox D. A Multi-scale CNN and Curriculum Learning Strategy for Mammogram Classification. In: Deep Learning in Medical Image Analysis and Multimodal Learning for Clinical Decision Support. Springer; 2017. p. 169–177.
55. Ribli D, Horváth A, Unger Z, Pollner P, Csabai I. Detecting and classifying lesions in mammograms with Deep Learning. arXiv preprint arXiv:170708401. 2017;.
56. Akselrod-Ballin A, Karlinsky L, Alpert S, Hashoul S, Ben-Ari R, Barkan E. A CNN based method for automatic mass detection and classification in mammograms. Computer Methods in Biomechanics and Biomedical Engineering: Imaging & Visualization. 2017;p. 1–8.
57. Carneiro G, Nascimento J, Bradley AP. Unregistered multiview mammogram analysis with pre-trained deep learning models. In: International Conference on Medical Image Computing and Computer-Assisted Intervention. Springer; 2015. p. 652–660.
58. Carneiro G, Nascimento J, Bradley AP. Automated Analysis of Unregistered Multi-View Mammograms With Deep Learning. IEEE Transactions on Medical Imaging. 2017;36(11):2355–2365.
59. Kooi T, Karssemeijer N. Classifying symmetrical differences and temporal change for the detection of malignant masses in mammography using deep neural networks. Journal of Medical Imaging. 2017;4(4):044501.
60. Ertosun MG, Rubin DL. Probabilistic visual search for masses within mammography images using deep learning. In: Bioinformatics and Biomedicine (BIBM), 2015 IEEE International Conference on. IEEE; 2015. p. 1310–1315.
61. Hwang S, Kim HE. Self-transfer learning for fully weakly supervised object localization. arXiv preprint arXiv:160201625. 2016;.
62. Akselrod-Ballin A, Karlinsky L, Alpert S, Hasoul S, Ben-Ari R, Barkan E. A region based convolutional network for tumor detection and classification in breast mammography. In: International Workshop on Large-Scale Annotation of Biomedical Data and Expert Label Synthesis. Springer; 2016. p. 197–205.
63. Carneiro NDG, Bradley AP. Automated Mass Detection from Mammograms using Deep Learning and Random Forest. 2016;.
64. Choukroun Y, Bakalo R, Ben-Ari R, Akselrod-Ballin A, Barkan E, Kisilev P. Mammogram Classification and Abnormality Detection from Nonlocal Labels using Deep Multiple Instance Neural Network. 2017;.
65. Zhu W, Xie X. Adversarial deep structural networks for mammographic mass segmentation. arXiv preprint arXiv:161205970. 2016;.
66. Dhungel N, Carneiro G, Bradley AP. A deep learning approach for the analysis of masses in mammograms with minimal user intervention. Medical image analysis. 2017;37:114–128.
67. Kisilev P, Sason E, Barkan E, Hashoul S. Medical image description using multi-task-loss CNN. In: International Workshop on Large-Scale Annotation of Biomedical Data and Expert Label Synthesis. Springer; 2016. p. 121–129.
68. Al-Masni M, Al-Antari M, Park J, Gi G, Kim T, Rivera P, et al. Detection and classification of the breast abnormalities in digital mammograms via regional Convolutional Neural Network. In: Engineering in Medicine and Biology Society (EMBC), 2017 39th Annual International Conference of the IEEE. IEEE; 2017. p. 1230–1233.
69. Platania R, Shams S, Yang S, Zhang J, Lee K, Park SJ. Automated Breast Cancer Diagnosis Using Deep Learning and Region of Interest Detection (BC-DROID). In: Proceedings of the 8th ACM International Conference on Bioinformatics, Computational Biology, and Health Informatics. ACM; 2017. p. 536–543.
70. Sun W, Tseng TLB, Zheng B, Qian W. A preliminary study on breast cancer risk analysis using deep neural network. In: International Workshop on Digital Mammography. Springer; 2016. p. 385–391.
71. Qiu Y, Wang Y, Yan S, Tan M, Cheng S, Liu H, et al. An initial investigation on developing a new method to predict short-term breast cancer risk based on deep learning technology. In: SPIE Medical Imaging. International Society for Optics and Photonics; 2016. p. 978521.
72. Fonseca P, Mendoza J, Wainer J, Ferrer J, Pinto J, Guerrero J, et al. Automatic breast density classification using a convolutional neural network architecture search procedure. In: Proc. of SPIE Vol. vol. 9414; 2015. p. 941428–1.
73. Fonseca P, Castañeda B, Valenzuela R, Wainer J. Breast Density Classification with Convolutional Neural Networks. In: Iberoamerican Congress on Pattern Recognition. Springer; 2016. p. 101–108.
74. Becker AS, Marcon M, Ghafoor S, Wurnig MC, Frauenfelder T, Boss A. Deep learning in mammography: diagnostic accuracy of a multipurpose image analysis software in the detection of breast cancer. Investigative Radiology. 2017;52(7):434–440.
75. Kallenberg M, Petersen K, Nielsen M, Ng AY, Diao P, Igel C, et al. Unsupervised deep learning applied to breast density segmentation and mammographic risk scoring. IEEE transactions on medical imaging. 2016;35(5):1322–1331.
76. Ahn CK, Heo C, Jin H, Kim JH. A Novel Deep Learning-based Approach to High Accuracy Breast Density Estimation in Digital Mammography. In: SPIE Medical Imaging. International Society for Optics and Photonics; 2017. p. 101342O–101342O.
77. Li S, Wei J, Chan HP, Helvie MA, Roubidoux MA, Lu Y, et al. Computer-aided assessment of breast density: comparison of supervised deep learning and feature-based statistical learning. Physics in Medicine & Biology. 2018;63(2):025005.
78. Wu N, Geras KJ, Shen Y, Su J, Kim S, Kim E, et al. Breast density

classification with deep convolutional neural networks. arXiv preprint arXiv:171103674. 2017;.
79. Thomaz RL, Carneiro PC, Patrocinio AC. Feature extraction using convolutional neural network for classifying breast density in mammographic images. In: Medical Imaging 2017: Computer-Aided Diagnosis. vol. 10134. International Society for Optics and Photonics; 2017. p. 101342M.
80. Mohamed AA, Luo Y, Peng H, Jankowitz RC, Wu S. Understanding Clinical Mammographic Breast Density Assessment: a Deep Learning Perspective. Journal of Digital Imaging. 2017;p. 1–6.
81. Mohamed AA, Berg WA, Peng H, Luo Y, Jankowitz RC, Wu S. A deep learning method for classifying mammographic breast density categories. Medical Physics. 2017;.
82. Qayyum A, Anwar SM, Awais M, Majid M. Medical image retrieval using deep convolutional neural network. Neurocomputing. 2017;.
83. Ahmad J, Sajjad M, Mehmood I, Baik SW. SiNC: Saliency-injected neural codes for representation and efficient retrieval of medical radiographs. PloS one. 2017;12(8):e0181707.
84. Umehara K, Ota J, Ishida T. Super-Resolution Imaging of Mammograms Based on the Super-Resolution Convolutional Neural Network. Open Journal of Medical Imaging. 2017;7(04):180.
